# Formulation of *Trichoderma* spp. encapsulated in alginate: potential for biofungicide with controlled conidial release

**DOI:** 10.1007/s11274-026-04989-9

**Published:** 2026-05-11

**Authors:** Thalesram Izidoro Pinotti, Vinicius de Paula Taffarel, Gabriel de Araújo Silva Cipriano, Ionnara Diogo Xavier, Yanka Manoelly dos Santos Gaspar, Luysa Valéria Leal Coêlho Ramos, Augusto Matias de Oliveira, Marcos Antonio Barbosa de Lima, Galba Maria de Campos-Takaki, Tiago de Oliveira Sousa, Thiago Pajeú Nascimento, Alice Maria Gonçalves Santos

**Affiliations:** 1 Campus Professora Cinobelina Elvas, Federal University of Piauí, Bom Jesus, 64900-000 PI Brazil; 2 University of Rio Verde, Rio Verde, 75901-970 GO Brazil; 3 Department of Biology, Federal Rural University of Pernambuco, Recife, 52171-900 PE Brazil; 4 Center for Research in Environmental Sciences, Department of Chemistry, Catholic University of Pernambuco, Recife, 50050-900 PE Brazil

**Keywords:** Biological Control, Biotechnology, Sodium alginate capsules, Conidial viability, PH-dependent release

## Abstract

The application of *Trichoderma* species as biological control agents requires stable formulations capable of preserving viability and ensuring consistent field efficacy. This study aimed to assess the stability and bio-efficacy of sodium alginate capsules containing conidia of *Trichoderma* species native to the Cerrado region of Piauí, Brazil. Five encapsulated species were evaluated for conidial viability after 15 months of storage, release profiles under different pH conditions, in vitro antagonistic activity against *Fusarium verticillioides*, and in vivo growth performance in different substrates. The capsules maintained structural integrity and viability above 1 × 10⁷ CFU mL⁻¹ for all species, with *Trichoderma* sp. 1 and *Trichoderma* sp. 2 exhibiting significant increases in conidial concentration over time. Conidial release was influenced by pH and exposure time, with the highest release observed at pH 7.0 for *T. longibrachiatum* and *T. koningiopsis*, while *Trichoderma* sp. 1 performed best at pH 5.0. In the antagonism assays, all species inhibited the growth of *F. verticillioides*, with *T. longibrachiatum* showing the strongest effect (45.97% inhibition). In in vivo bioassays, efficient colonization was observed across all tested substrates (soil, soil mixed with commercial substrate, and commercial substrate alone), with visible sporulation within 72 h. *Trichoderma* sp. 1 demonstrated the highest stability across substrates, whereas *T. koningiopsis* grew exclusively on commercial substrate. Collectively, these findings demonstrate that sodium alginate encapsulation effectively preserves the viability, structural stability, and biocontrol functionality of *Trichoderma* spp., underscoring its potential for development into commercial formulations suitable for diverse agricultural systems.

**Graphical abstract:**

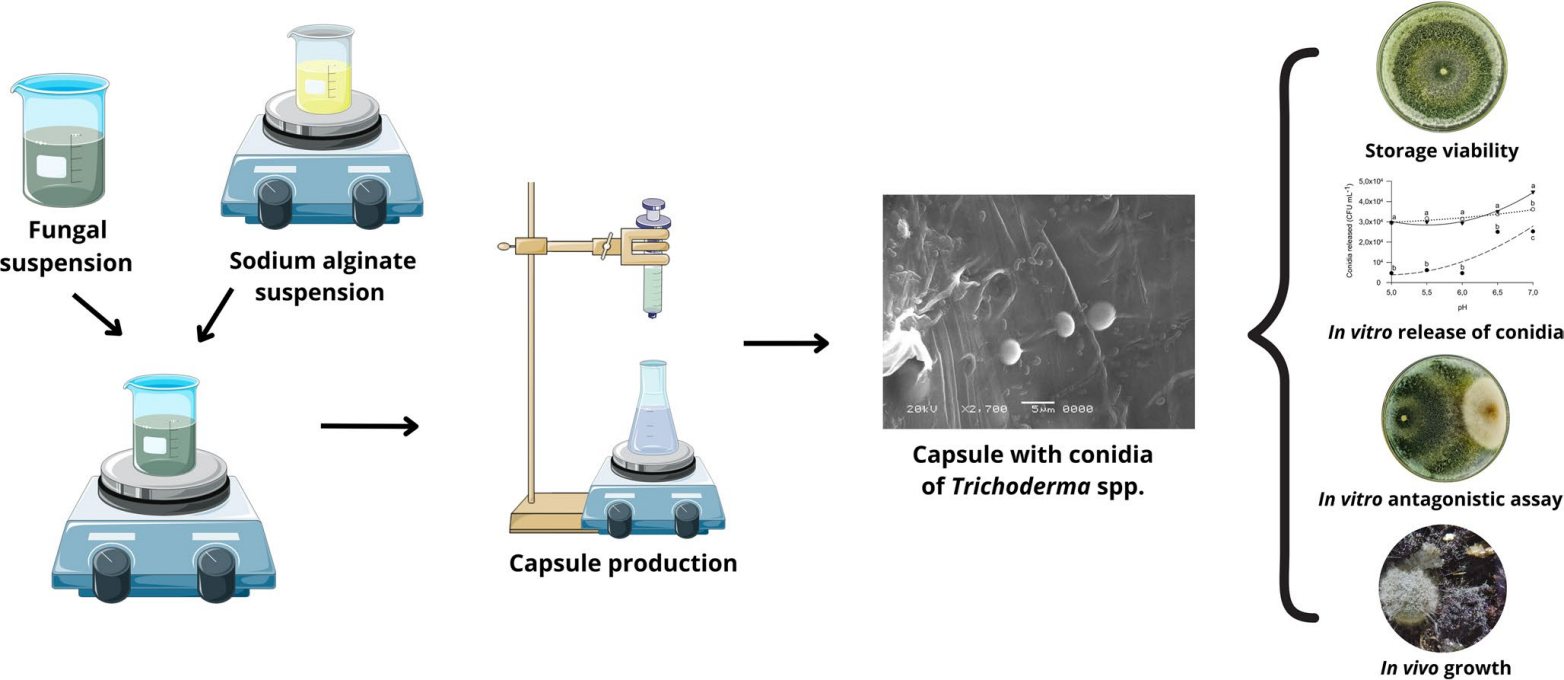

**Supplementary Information:**

The online version contains supplementary material available at 10.1007/s11274-026-04989-9.

## Introduction

Fungal diseases caused by soil-borne pathogens lead to substantial losses in both agricultural production and natural ecosystems (Pérez-Pizá et al. 2024; Panwar et al. 2025). Chemical fungicides are commonly employed as an effective means of phytosanitary management (Anani et al. 2020; McLaughlin et al. 2023). However, their indiscriminate and excessive use poses serious drawbacks, including risks to human health, environmental contamination, and biodiversity loss (Hazra et al. 2023; Zhu et al. 2024). In addition, prolonged exposure of phytopathogenic fungi to these compounds can promote the development of resistance, thereby progressively diminishing their efficacy (Hazra et al. 2023; Fenta and Mekonnen 2024).

The use of beneficial soil microorganisms represents a viable and environmentally safe alternative to synthetic chemical fungicides (Ben Khedher et al. 2021). Beyond disease suppression, these microorganisms play a crucial role in promoting ecosystem stability (Asghar et al. 2024). *Trichoderma harzianum* and *Trichoderma viride* are among the most widely employed species for antagonizing plant pathogens (Timmanna Bhat and Shishupala 2024). Nevertheless, the exploration and application of novel *Trichoderma* species for biological control remain essential. Species isolated from plants in the Cerrado region of Piauí, Brazil, exhibit promising biotechnological potential for disease management, either through direct competition or the synthesis of bioactive secondary metabolites (Silva et al. 2021; Morais et al. 2022).

Various strategies have been developed to formulate products derived from fungal biomass (Martinez et al. 2023). However, these formulations still face significant challenges, including low viability and stability, which hinder the microorganisms’ capacity to establish and fully express their antagonistic potential against phytopathogens. Effective formulations must provide adequate protection to ensure both storage stability and successful field application of the biofungicide. Conidial microencapsulation has emerged as a promising technique to mitigate adverse abiotic factors while enhancing the survival, establishment, and antagonistic activity of the microorganism (Saberi-Riseh et al. 2021).

Microcapsules are typically generated from liquid droplets through either dripping or emulsification processes. A wide range of polymers, including alginate, starch, pectin, cellulose, agar, and chitosan, are commonly employed in encapsulation (Saberi-Riseh et al. 2021). Alginate has been extensively used to encapsulate *Trichoderma* spp. conidia, supporting both the viability of formulations (Adzmi 2021; Pinotti et al. 2026) and the expression of antagonistic traits (Locatelli et al. 2018; Pinotti et al. 2025). Owing to its biodegradable and non-toxic properties, sodium alginate is widely recognized as a suitable carrier material. Moreover, its ability to form cross-links with calcium ions makes it an optimal matrix for encapsulating cells, drugs, and other bioactive molecules, thereby facilitating their protection, controlled administration, and sustained release (Rodrigues et al. 2020; Martínez-Cano et al. 2022).

In addition to the choice of carrier material, product viability is a critical factor to be considered. It is essential to assess how prolonged storage and the release dynamics of encapsulated conidia influence fungal growth. Moreover, there remains a notable gap in research regarding capsule performance in soil and their biofungicidal activity, particularly under field conditions. Addressing these aspects is fundamental to optimizing both the production and practical application of encapsulated formulations as biofungicides. Accordingly, the present study aimed to evaluate the stability and bio-efficacy of sodium alginate capsules containing *Trichoderma* sp. conidia from the Piauí Cerrado, providing a basis for their potential use in phytosanitary management.

## Materials and methods

### Origin of *Trichoderma* capsules

Five *Trichoderma* species - *T. longibrachiatum* (UFPI02), *T. koningiopsis* (UFPI03), *Trichoderma* sp.1 (UFPI04), *Trichoderma* sp.2 (UFPI13), and *T. orientale* (UFPI14) - were obtained from the biocontrol culture collection maintained at the Phytopathology Laboratory of the Federal University of Piauí (UFPI), Professora Cinobelina Elvas Campus (CPCE), Bom Jesus, Piauí, Brazil. Encapsulation was carried out in November 2023 using the ionic gelation method, in which a sodium alginate suspension containing fungal propagules adjusted to a final concentration of 1 × 10^7^ CFU mL^− 1^ was dripped into a calcium chloride (CaCl₂) solution (dos Santos et al. 2015). The capsules were stored in amber glass containers at 30 °C.

### Capsule stabilities

Following capsule formulation, samples were stored for 10 and 15 months at room temperature to evaluate the long-term stability and viability of the isolates. After each storage period, capsules were individually transferred to Petri dishes containing potato dextrose agar (PDA) and incubated in a BOD (Biochemical Oxygen Demand) growth chamber at 28 °C. Fungal growth characteristics and conidial production were assessed, with concentrations expressed as colony-forming units per milliliter (CFU mL^− 1^). For this purpose, a fungal suspension was prepared by adding 20 mL of distilled water per plate, followed by scraping with a glass rod and filtration through a triple layer of sterile gauze. Conidia were counted using a Neubauer chamber.

The results were compared with viability data from capsules stored for five months, as well as with the initial conidial concentration prior to encapsulation, previously reported by Pinotti et al. (2025). Capsules were considered viable when fungal growth yielded concentrations greater than 1 × 10^7^ CFU mL^− 1^. The experiment was conducted with four replicates under a completely randomized design (CRD).

### Scanning electron microscopy (SEM)

The surface morphology of the capsules and the presence of conidia were examined using scanning electron microscopy. Capsules representative of the overall morphology of the samples were selected for analysis, lyophilized for 48 h, mounted on aluminum stubs, and sputter-coated with a 10 nm layer of gold. Samples were then observed under high vacuum conditions in a JEOL JSM 5600 LV scanning electron microscope operated at 20 kV.

### Release profile of encapsulated conidia in different pH solutions

To assess the conidial release rate of encapsulated species under different pH conditions in vitro, capsules stored for 15 months were immersed in phosphate buffer solutions adjusted to pH 5.0, 5.5, 6.0, 6.5, and 7.0, simulating the rhizospheric environment of maize fields. The assay followed a protocol adapted from Santos-Díaz et al. (2022), in which ten capsules of each species were subjected to orbital shaking at 150 rpm. Aliquots were collected after 1 h, 5 h, and 24 h, mixed with 0.1% Tween solution, and plated on PDA medium. Plates were incubated at 28 °C, and colony counts performed after 24 h were used to quantify conidial release (CFU mL^− 1^).

The experiment was conducted with four replicates in a 5 × 3 factorial design (pH x time), arranged in CRD.

### In vitro antagonistic potential against *Fusariumverticillioides*

*Fusarium verticillioides* isolate was obtained from the fungal collection of the Phytopathology Laboratory at UFPI-CPCE, originally recovered from maize stems. Genetic characterization was performed by sequencing the translation elongation factor 1-α (EF1-α) and the second largest subunit of RNA polymerase II (RPB2) genes. The resulting sequences were deposited in the GenBank database of the National Center for Biotechnology Information (NCBI) under accession numbers PZ309777 and PZ309778. The isolate was cultured under controlled conditions on 80-mm Petri plates containing PDA medium and incubated for seven days at 28 °C.

The pairing assay was performed in Petri dishes containing PDA. Five-millimeter-diameter disks previously colonized with *F. verticillioides* were placed at one end of the plate, while 15-month-old *Trichoderma* capsules were placed at the opposite end. Plates containing only *F. verticillioides* served as controls. All plates were incubated for seven days in a BOD chamber under a 12-h photoperiod, and colony growth was measured every 24 h. The experiment followed a completely randomized design with four replicates.

The variables analyzed included the final colony diameter (FCD) of *F. verticillioides*, the percentage of growth inhibition (PGI), and the degree of antagonism according to the scale of Bell (1982). *Trichoderma* species were classified as antagonistic when the mean score was ≤ 2 and non-antagonistic when the mean score was ≥ 3. The final colony diameter of *F. verticillioides* was determined with a digital caliper on the seventh day of evaluation, and the radius was squared to calculate colony area. Inhibition values were calculated according to the following equation:

1 $$\:\begin{array}{cc}&\:\mathrm{P}\mathrm{G}\mathrm{I}\left(\mathrm{\%}\right)=\frac{\mathrm{C}-\mathrm{T}}{\mathrm{C}}\times\:100\end{array}$$

where C = colony growth in the control and T = colony growth in the treatment.

### In vivo growth assay

#### Soil preparation and substrate

Soil for the in vivo bioassay was collected from the UFPI maize experimental field, CPCE. The commercial substrate used was Carolina Soil^®^, consisting of sphagnum peat, expanded perlite, expanded vermiculite, limestone, organomineral fertilizer (PFT), and rice hulls. Both the soil and the commercial substrate were sterilized by autoclaving at 120 °C for 1 h and stored in the dark. After 24 h, the materials were autoclaved again under the same conditions to ensure complete sterilization and subsequently used in the experiments.

#### Growth assay

Gerboxes (11.5 × 11.5 × 3.5 cm) were filled with three different media: (i) 100 g of soil, (ii) 100 g of soil supplemented with 2 g of commercial substrate, and (iii) 30 g of commercial substrate alone. The boxes were incubated at 28 °C in the dark for 24 h. After this period, substrates were moistened with sterile water to 60% of their water-holding capacity. Subsequently, 2 mL of a sterile nutrient solution containing 0.5% (w/v) yeast extract and 1% (w/v) glucose was added.

For each treatment, three capsules containing *Trichoderma* spp. were placed on the soil surface. Control boxes containing only the growth media (soil, soil + commercial substrate, or commercial substrate) were also prepared. The boxes were sealed and incubated in the dark at 28 °C for 24 h. After this period, boxes containing capsules were exposed to a 12 h light/12 h dark photoperiod while maintaining the same temperature. The experiment was arranged in CRD with four replicates.

Initial growth of *Trichoderma* spp. was visually assessed under a magnifying glass at 24, 48, and 72 h post-inoculation, verifying fungal emergence from the capsules. Ten days after inoculation, samples from each treatment were collected for serial dilution to confirm the growth of encapsulated *Trichoderma*, while control samples were used to check for contaminant microorganisms.

For serial dilution, 1 g of substrate was suspended in 9 mL of sterile distilled water (SDW), homogenized, and serially diluted to 10^− 3^ following Pepper and Gerba (2005). Aliquots from each dilution were plated onto PDA and incubated in a BOD chamber at 28 °C. After seven days, *Trichoderma* colonies were reisolated.

Mycelial discs (5 mm diameter) from reisolated colonies were transferred to PDA plates and incubated at 28 °C for seven days. Conidial concentration was quantified as previously described and compared with the reference value established during the capsule viability assessment after 15 months of storage, to evaluate the effect of different substrates on *Trichoderma* sporulation.

### Statistical analysis

Data were tested for normality (Shapiro-Wilk) and homogeneity of variance (Bartlett) at a 5% significance level, followed by analysis of variance (ANOVA). When significant differences were detected, means were grouped using the Scott-Knott test at a 5% probability level. Analyses were performed in R v.4.2.1, and graphs were generated using SigmaPlot v.16.0.

## Results

### Capsule stability

After 15 months of storage at room temperature (30 °C), the capsules remained structurally intact and opaque, with coloration ranging from whitish to greenish depending on the encapsulated species (Fig. S1). No signs of contamination were detected. The texture remained homogeneous, with no evidence of excessive dryness or brittleness. Capsule surfaces were smooth, showing no visible cracks or deformation, and mechanical resistance was preserved, with no signs of disintegration upon handling.

**Fig. 1:**
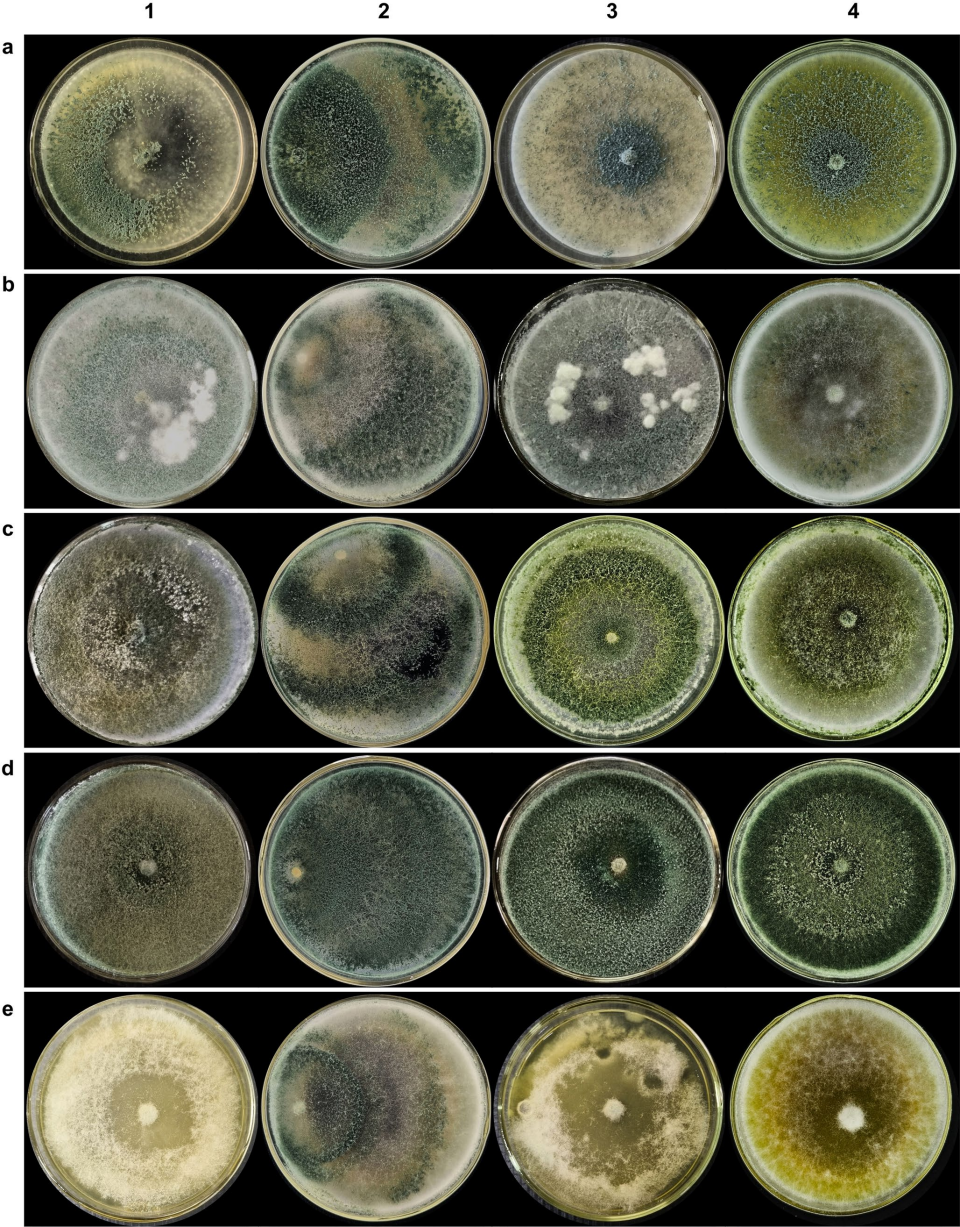
Growth of *T. longibrachiatum* (**A**), *T. koningiopsis* (**B**), *Trichoderma* sp.1 (**C**), *Trichoderma* sp.2 (**D**), and *T. orientale* (**E**) before (1) and after 5 (2), 10 (3), and 15 (4) months of encapsulation

**Fig. 2:**
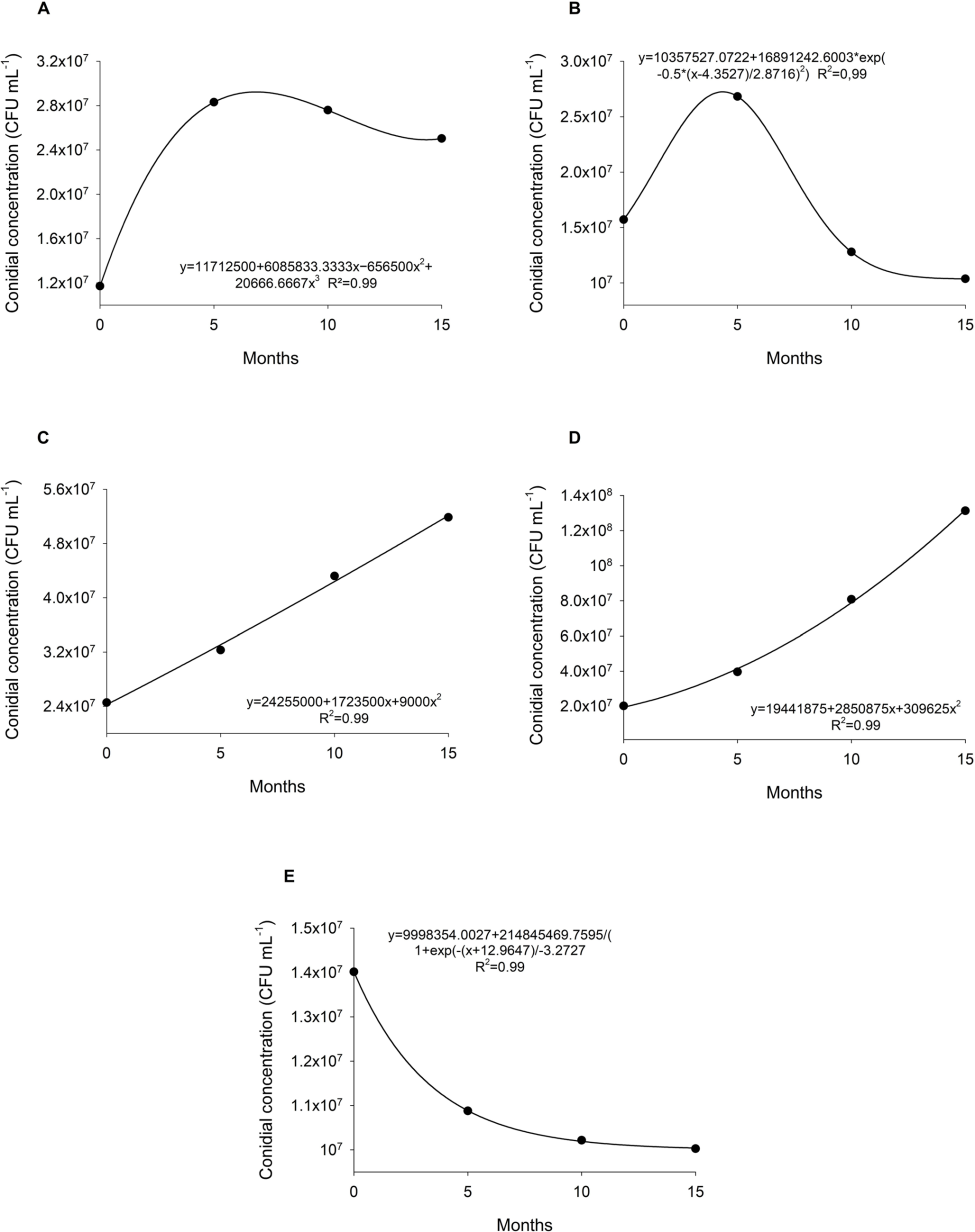
Conidia concentration of *T. longibrachiatum* (**A**), *T. koningiopsis* (**B**), *Trichoderma* sp. 1 (**C**), *Trichoderma* sp. 2 (**D**) and *T. orientale* (**E**) during storage time

All *Trichoderma* species exhibited vigorous growth, forming well-developed colonies with homogeneous texture and pigmentation ranging from whitish to olive green to deep green (Fig. 1). After five months of storage, colonies retained their typical morphology, with only slight variations in pigmentation (Fig. 1-2). However, after ten months, *T. longibrachiatum* displayed mycelial growth closer to the culture medium surface, differing from the growth pattern observed both before and after five months of encapsulation (Fig. 1A3).

**Fig. 3:**
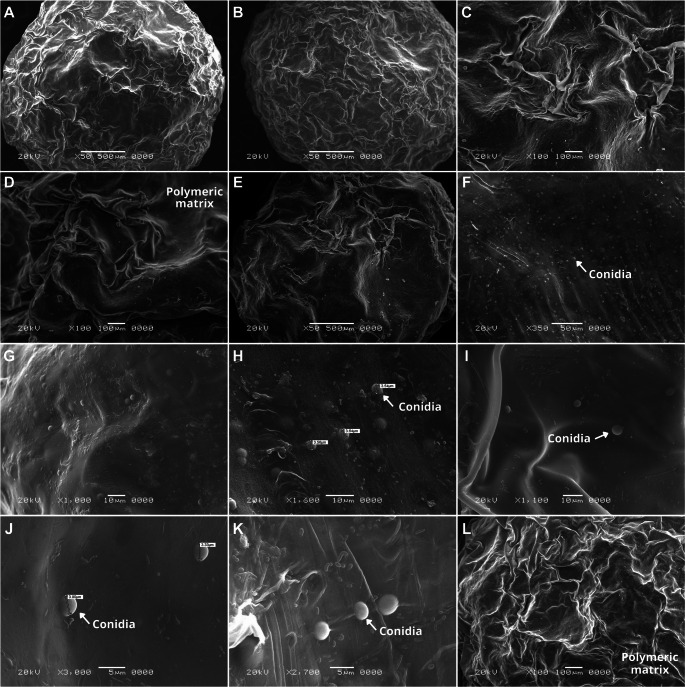
Representative SEM micrographs of capsules containing *Trichoderma* conidia after freeze-drying. External capsule structures (**A-F, L**) and internal capsule structures showing immobilized conidia (**G-K**)

**Fig. 4:**
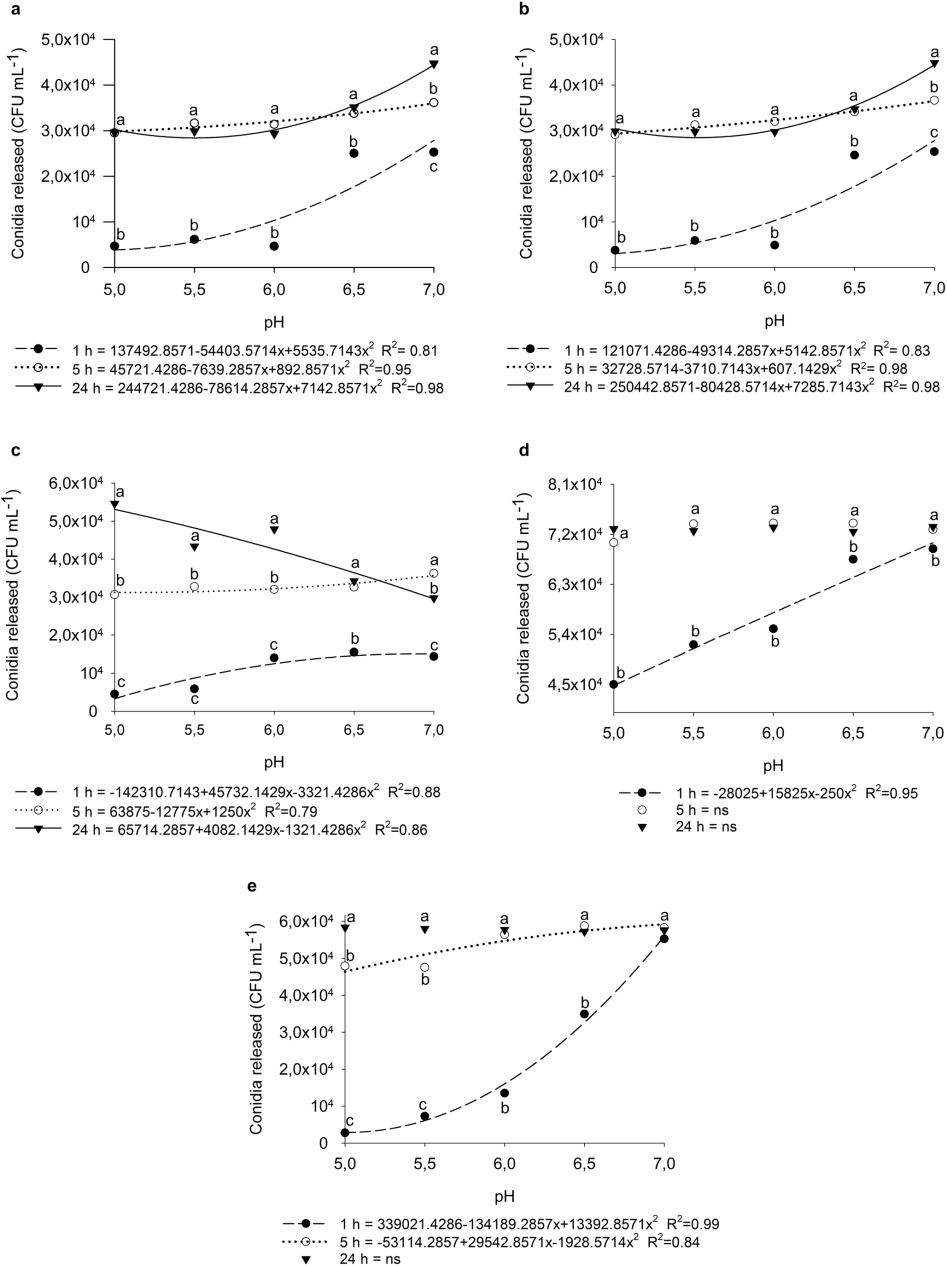
Release of encapsulated conidia of *T. longibrachiatum* (**A**), *T. koningiopsis* (**B**), *Trichoderma* sp. 1 (**C**), *Trichoderma* sp. 2 (**D**), and *T. orientale* (**E**) as a function of pH after 1, 5, and 24 h of shaking. The curves represent the regression models fitted for each evaluation time. Means followed by the same letter within each pH level not differ among shaking times according to the Scott-Knott test at the 5% probability level. ns = not significant

After fifteen months of storage, all species maintained growth, although with more pronounced variations in pigmentation and mycelial development (Fig. 1-4). Colonies of *Trichoderma* sp. 1 (Fig. 1C4) and *Trichoderma* sp. 2 (Fig. 1D4) exhibited a more intense greenish coloration compared with the initial plating. Some colonies developed deeper green tones, whereas others displayed yellowish hues. *T. orientale* (Fig. 1E) showed a more irregular morphology relative to the other species. Despite these variations, no signs of contamination were observed in any of the plates.

Regarding conidial concentration, all species retained capsule viability above 1 × 10^7^ CFU mL^− 1^ before and after storage (Table S1). In some cases, conidial concentrations after storage exceeded pre-encapsulation values. Significant variation in conidial concentrations was observed among the inoculated species over the 15-month storage period (Table S1). Growth curves were fitted to different mathematical models: a cubic function for *T. longibrachiatum* (A), a cumulative Gaussian function for *T. koningiopsis* (B), a quadratic function for *Trichoderma* sp. 1 (C) and *Trichoderma* sp. 2 (D), and a sigmoidal function for *T. orientale* (E).

Comparison of the present data (10 and 15 months) with the five-month evaluation previously reported by Pinotti et al. (2025) revealed distinct patterns among species. *T. longibrachiatum* showed a marked increase in conidial concentration between the initial and five-month evaluations, rising from 1.2 × 10^7^ to 2.8 × 10^7^ CFU mL^− 1^, after which values remained stable (Fig. 2A). A similar trend was observed for *T. koningiopsis*, which increased from 1.5 × 10^7^ to 2.5 × 10^7^ CFU mL^− 1^ in five months, followed by a significant decline; however, concentrations remained above 1 × 10^7^ CFU mL^− 1^ (Fig. 2B).

In contrast, *Trichoderma* sp. 1 and *Trichoderma* sp. 2 benefited from longer storage, with progressive increases in conidial concentration during plate growth. *Trichoderma* sp. 1 increased from 2.4 × 10^7^ to 5.1 × 10^7^ CFU mL^− 1^ (Fig. 2C), while *Trichoderma* sp. 2 showed a remarkable rise from 2.0 × 10^7^ to 1.310^8^ CFU mL^− 1^ after 15 months (Fig. 2D). Conversely, *T. orientale* exhibited a significant reduction (*p* < 0.0001), decreasing from 1.4 × 10^7^ to 1.0 × 10^7^ CFU mL^− 1^, although concentrations remained stable between the 10- and 15-month evaluations (Fig. 2E).

### Scanning electron microscopy

Initially, the capsules were spherical, regular, opaque, and exhibited a coloration ranging from transparent to greenish. After drying, however, the capsules of the five *Trichoderma* species developed irregular and wrinkled surfaces, along with noticeable shrinkage due to dehydration (Fig. 3A-C).

Examination of the internal structure confirmed the effective immobilization of *Trichoderma* conidia within the polymer matrix. The conidia were dispersed across different regions of the capsules, verifying their successful incorporation (Fig. 3F-H). Scaling analysis further demonstrated that the conidia retained their morphological integrity, with sizes ranging from 3.33 to 3.90 μm. Moreover, the absence of conidia on the external capsule surface indicated efficient encapsulation, providing protection against abiotic stress factors.

### Release profile of encapsulated conidia in different pH solutions

A significant interaction was observed between pH solutions and agitation times for all encapsulated species (Tables S2-S6). For all parameters evaluated, data were best described by a quadratic polynomial model. As pH increased, there was a general trend toward greater conidial release during the first hour of agitation across all species, a pattern that was also observed at 5 and 24 h for *T. longibrachiatum* and *T. koningiopsis* (Fig. 4A and 4B). For *Trichoderma* sp. 1 and *T. orientale*, this response was evident only after 5 h of agitation (Fig. 4).

For *T. longibrachiatum* and *T. koningiopsis*, the highest conidial release occurred after 24 h in the pH 7.0 solution, reaching approximately 4 × 10^4^ CFU mL^− 1^ (Fig. 4A and 4B). In contrast, *Trichoderma* sp. 1 displayed a distinct pattern, with peak release at pH 5.0 followed by a marked decline at pH 7.0 after 24 h (Fig. 4C). *Trichoderma* sp. 2 showed no significant differences among pH treatments at either 5-24 h, and complete release of encapsulated conidia had already occurred within the first hour of agitation at pH 7.0 (Fig. 4D). Conversely, in *T. orientale*, conidial release stabilized after 24 h, reaching maximum levels across all tested pH values (Fig. 4E).

For *T. longibrachiatum* and *T. koningiopsis*, no significant differences in conidial release were observed within the pH range of 5.0 to 6.5 at either 5-24 h of shaking, with differences detected only at pH 7.0 (Fig. 4A and 4B). In contrast, *Trichoderma* sp. 1 exhibited significant differences among all shaking times within the pH range of 5.0 to 6.0 (Fig. 4C).

For *Trichoderma* sp. 2 and *T. orientale*, no significant differences were detected among shaking times at pH 7.0 (Fig. 4D and 4E). Within the pH range of 5.0 to 6.5, *Trichoderma* sp. 2 also showed no differences between 5 and 24 h. However, for *T. orientale*, significant differences were observed across the three shaking times at pH 5.0 and 5.5, with higher conidial release occurring at 24 h (Fig. 4E).

### In vitro antagonistic potential against *F. verticillioides*

**Fig. 5:**
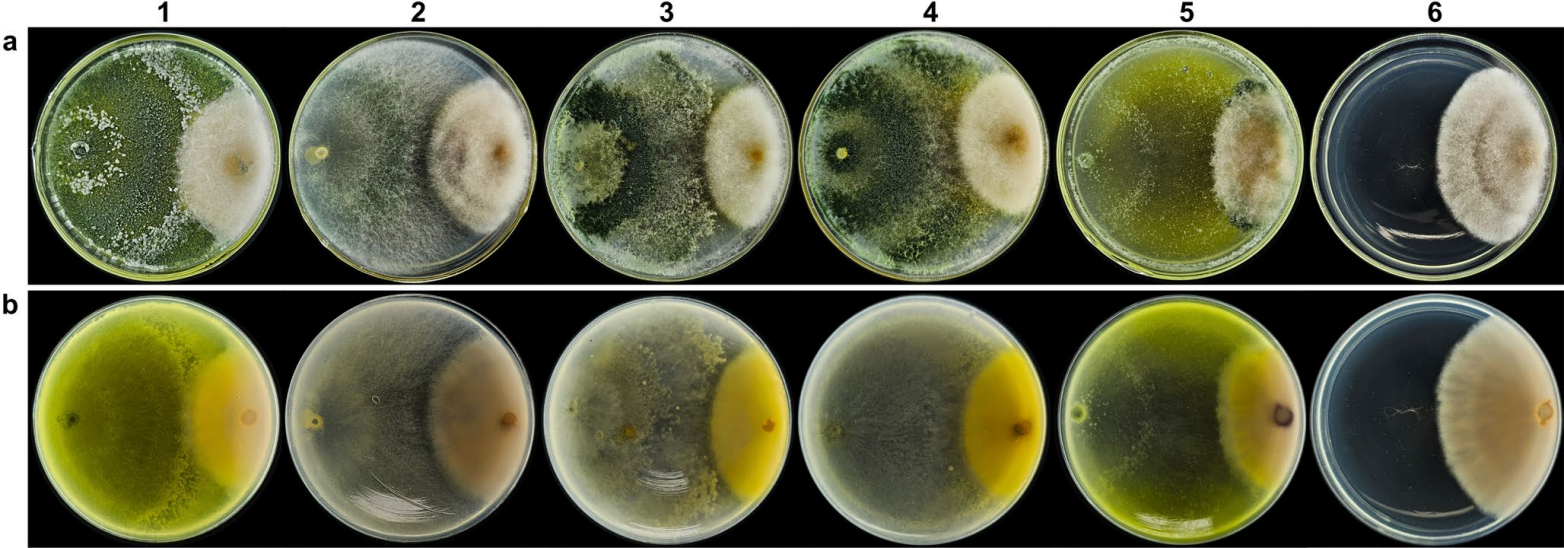
Antagonistic effect of *T. longibrachiatum* (1), *T. koningiopsis* (2), *Trichoderma* sp. 1 (3), *Trichoderma* sp. 2 (4), and *T. orientale* (5) against *F. verticillioides* (6). Front view (**A**) and back view (**B**) of the plate, seven days after inoculation

*Trichoderma* species demonstrated antagonistic activity against *F. verticillioides* (Fig. 5). On average, the tested isolates scored four on the Bell scale, indicating strong antagonistic interactions. *T. longibrachiatum* and *T. orientale* stood out, each achieving a score of five, characterized by complete overgrowth of the pathogen colony (Fig. 5A1 and 5A5).

**Fig. 6:**
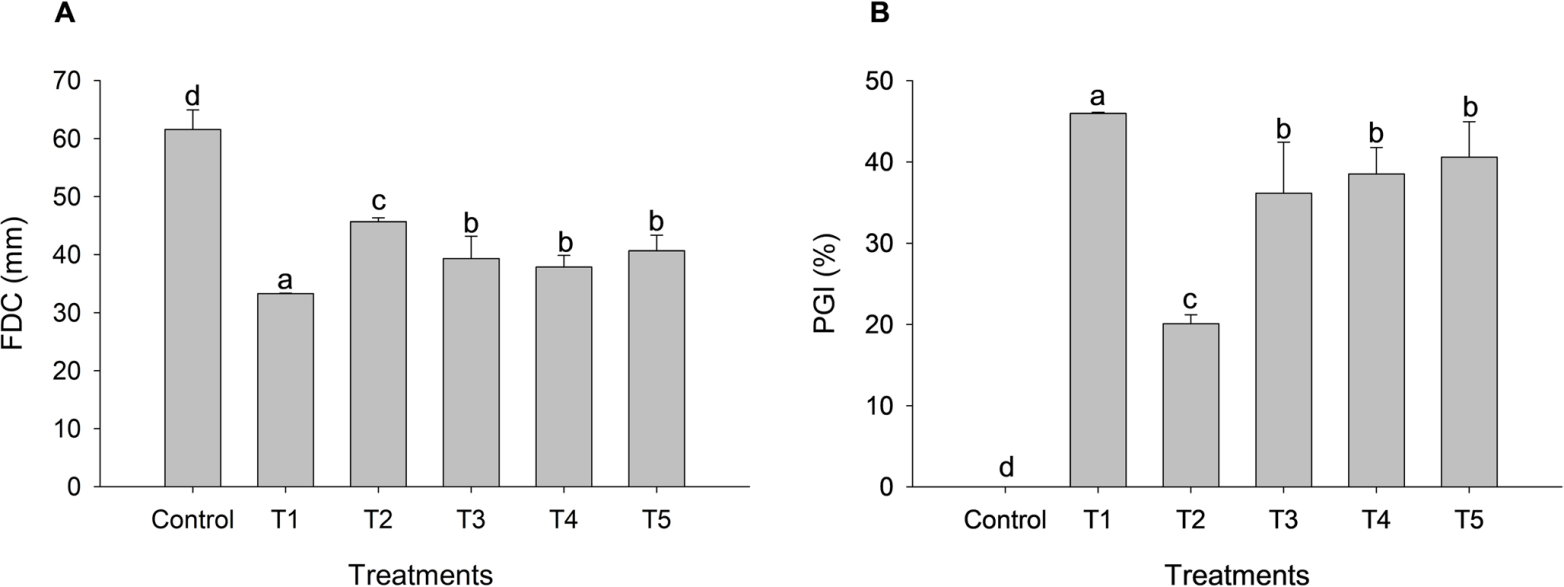
Antagonism of microencapsulated *T. longibrachiatum* (T1), *T. koningiopsis* (T2), *Trichoderma* sp. 1 (T3), *Trichoderma* sp. 2 (T4), and *T. orientale* (T5) on the final colony diameter (**A**) and percentage of growth inhibition (**B**) of *F. verticillioides*. Means followed by the same letter do not differ significantly according to the Scott-Knott test at 5% probability

For the variables FCD and PGI, significant differences were observed among treatments (Table S7). *T. longibrachiatum* resulted in the smallest final colony diameter of *F. verticillioides*, differing statistically from *Trichoderma* sp. 1, *Trichoderma* sp. 2, and *T. orientale*. Although *T. koningiopsis* promoted the least reduction in FCD, it still differed significantly from the control (Fig. 6A). The highest percentage of mycelial growth inhibition was observed for *T. longibrachiatum* (45.97%), followed by *T. orientale* (40.58%), *Trichoderma* sp. 2 (38.58%), and *Trichoderma* sp. 1 (36.15%) (Fig. 6B).

### In vivo growth assay

**Fig. 7:**
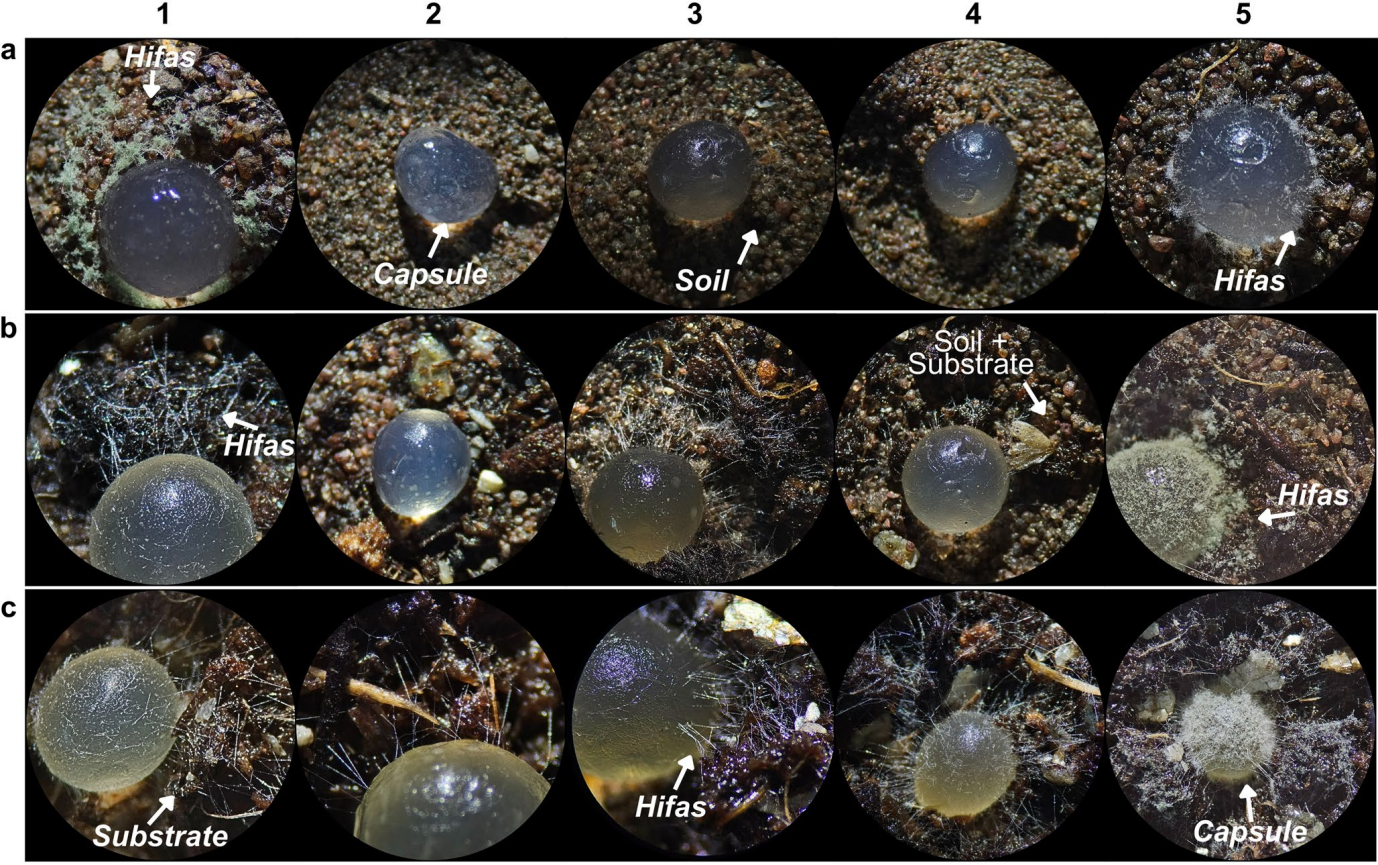
Growth of encapsulated *T. longibrachiatum* (1), *T. koningiopsis* (2), *Trichoderma* sp. 1 (3), *Trichoderma* sp. 2 (4), and *T. orientale* (5) at 72 h after inoculation in (**A**) soil, (**B**) soil + commercial substrate, and (**C**) commercial substrate

The conidia of the encapsulated species initiated germination approximately 24 h after being introduced into the growth medium, with hyphae emerging from the capsules and extending toward the respective substrates. After 72 h, greenish spots became visible (Fig. 7A1 and 7B5), indicating sporulation and the onset of the reproductive cycle.

Encapsulated *T. longibrachiatum*, *Trichoderma* sp. 1, *Trichoderma* sp. 2, and *T. orientale* were able to grow on all evaluated media (Fig. 7), although their growth behavior varied depending on the substrate. In contrast, *T. koningiopsis* displayed growth only on the commercial substrate (Fig. 7C2).

Serial dilution analyses confirmed successful colonization by all inoculated species across all treatments, with consistently high recovery rates and no evidence of contaminating microorganisms in either the treatments or the control. Moreover, no noticeable morphological changes were observed in the hyphae or conidia of reisolated colonies across the different culture systems (Fig. S2).

**Fig. 8:**
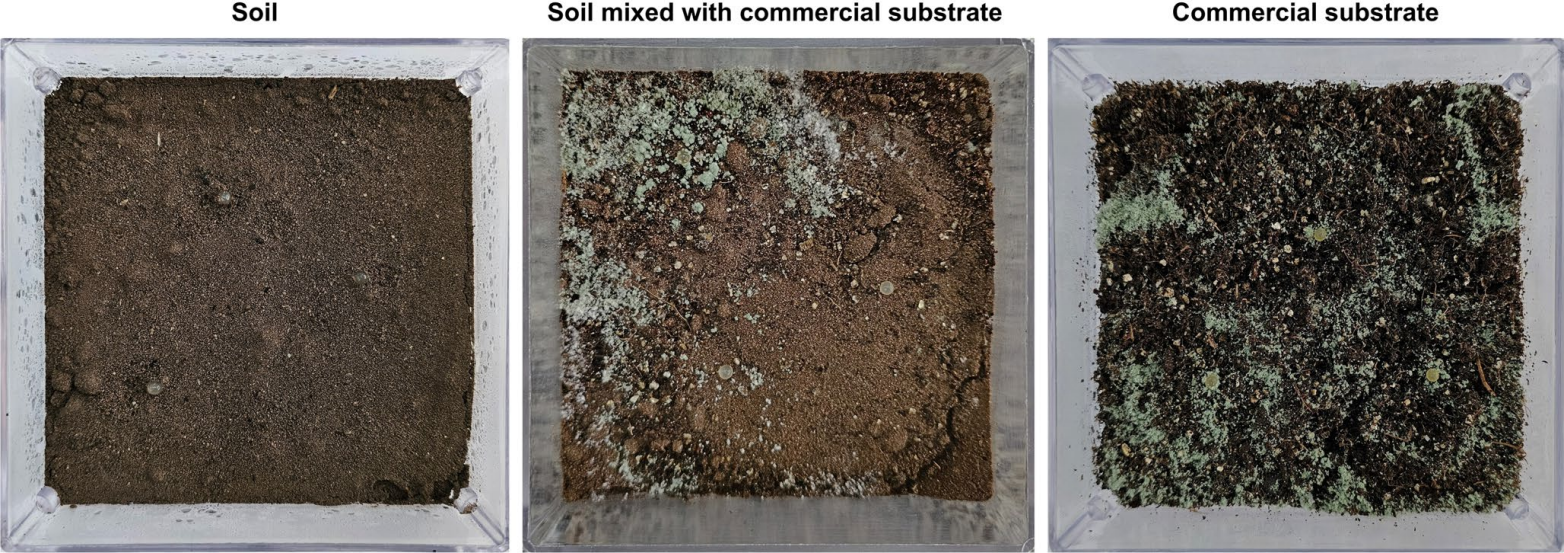
Representative growth of *Trichoderma* capsules in different culture media

Capsule dissolution occurred more rapidly in soil compared with the other treatments, followed by soil + commercial substrate and commercial substrate alone. Soil moisture appeared to accelerate the dissolution process. In contrast, treatments containing commercial substrate promoted more robust colonization, evidenced by greater mycelial development and more intense sporulation, compared with treatments containing soil alone (Fig. 8).

The conidial concentration of reisolated fungi after exposure to the different treatments exhibited species-specific patterns (Table S8). All species that successfully colonized the tested substrates remained viable after reisolation, showing concentrations above 1 × 10⁷ CFU mL⁻¹, with the exception of *T. koningiopsis*, which was reisolated only from the commercial substrate treatment.

**Fig. 9:**
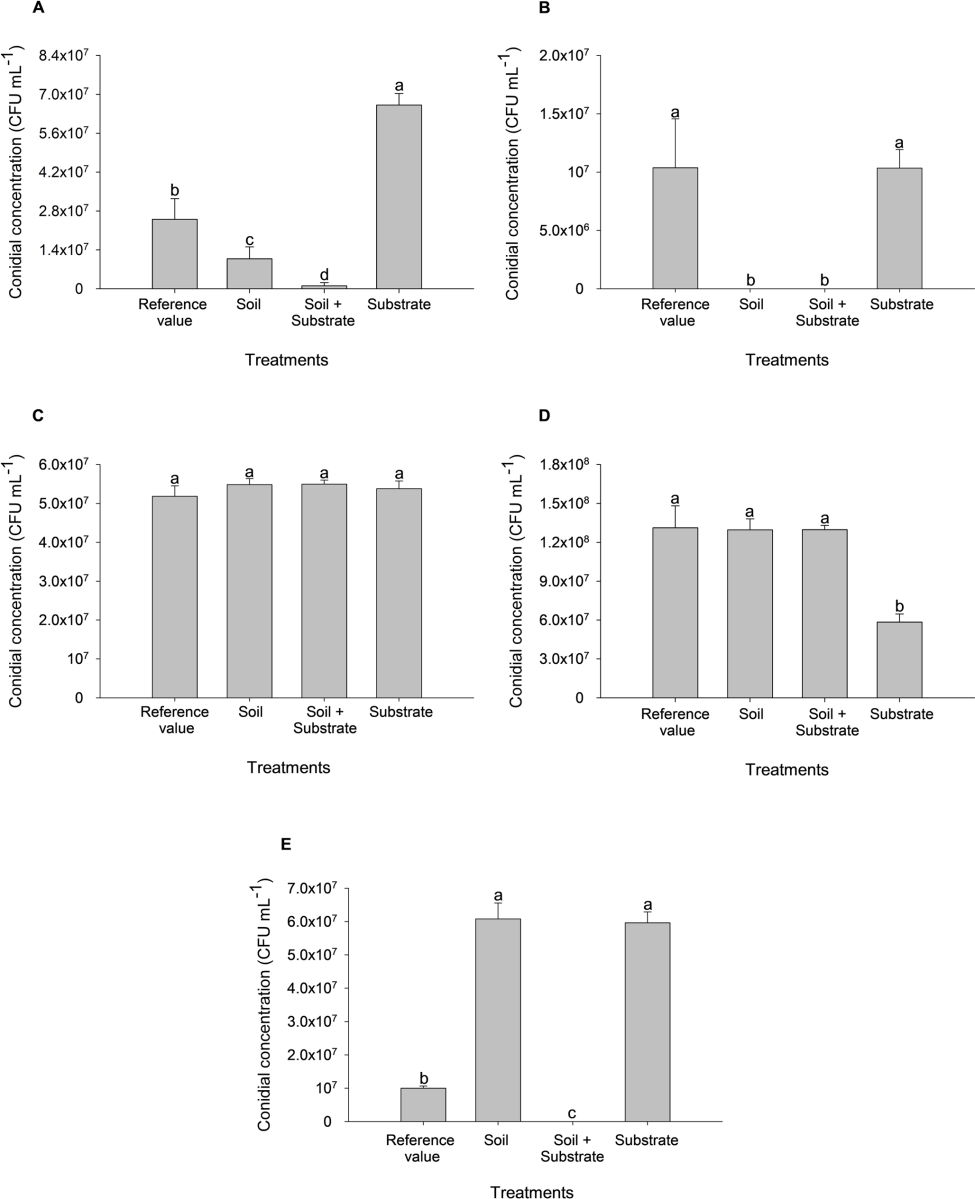
Conidial concentration of *T. longibrachiatum* (**A**), *T. koningiopsis* (**B**), *Trichoderma* sp. 1 (**C**), *Trichoderma* sp. 2 (**D**), and *T. orientale* (**E**) re-isolated from different growth media. Means followed by the same letter do not differ significantly according to the Scott-Knott test at 5% probability

*Trichoderma* sp. 1 proved to be the most stable species, showing no difference among treatments compared with the reference value, maintaining an average concentration of 5 × 10⁷ CFU mL⁻¹ (Fig. 9C). By contrast, *T. longibrachiatum* reached the highest concentration in the commercial substrate treatment (6 × 10⁷ CFU mL⁻¹), which was significantly higher than both the other treatments and the reference value (1 × 10⁷ CFU mL⁻¹) (Fig. 9A).

For *T. koningiopsis*, growth was restricted to the commercial substrate treatment, where its conidial concentration did not differ significantly from the reference (Fig. 9B). *Trichoderma* sp. 2 showed the highest concentrations in soil and soil + commercial substrate treatments, though not statistically different from the reference value; however, the treatment containing only soil resulted in a lower concentration compared with both the reference and other treatments (Fig. 9D).

Although *T. orientale* was able to colonize the soil + commercial substrate treatment, it did not produce sporulation upon reisolation. In contrast, treatments containing only soil or only commercial substrate promoted a significant increase in conidial concentration, reaching an average of 5.5 × 10⁷ CFU mL⁻¹, higher than the reference value (Fig. 9E).

## Discussion

### Capsule stability

The maintenance of conidial viability over time is a critical factor for the practical application of biological control agents, as propagule survival commonly declines during storage across a wide range of temperatures (Fu and Chen 2011). In the present study, fungal concentrations varied among species; however, all encapsulated isolates retained biologically relevant viability levels throughout the storage period (Table S1). For *Trichoderma* sp. 1 and *Trichoderma* sp. 2, encapsulation resulted in enhanced growth and higher CFU counts, likely reflecting reduced physiological stress during storage. This protective effect may be influenced by physicochemical properties of the capsules, including bead size, which is known to affect diffusion processes, internal moisture balance, and metabolic activity within encapsulated systems. Although bead size and related parameters were not systematically evaluated in this study, their potential contribution to the observed viability patterns should be considered in future investigations focused on optimizing formulation performance and storage stability.

Furthermore, it was confirmed that alginate capsules preserve the viability of encapsulated conidia even under elevated average temperatures (33 °C) after several months of storage. This finding contrasts with some reports in the literature suggesting that lower temperatures (5-8 °C) are optimal for conservation, ensuring viability for three to ten months (Lotfalinezhad et al. 2024). Thus, the possibility of storage at room temperature becomes attractive, as it may reduce production costs.

This protection is attributed to the ability of sodium alginate to form stable three-dimensional hydrogels in the presence of divalent ions such as calcium (Ca²⁺) (Zhang et al. 2020). These hydrogels provide a favorable microenvironment for conidia by restricting excessive gas exchange and water loss, while allowing selective diffusion of nutrients and oxygen at levels sufficient to maintain basal metabolism. This supports dormancy maintenance and extends fungal viability for months or even years without significant loss of biological activity. Bhai (2020) reported that *Trichoderma* conidia encapsulated in alginate beads remained viable for more than six years at room temperature, exhibiting rapid germination after rehydration.

In the present study, the observed differences between encapsulated *Trichoderma* isolates are likely associated not only with the intrinsic biological characteristics of each isolate, such as conidial morphology and metabolic activity, but also with their specific interactions with the alginate matrix. Structural and biochemical properties of conidia can influence how they interface with the polymer network, affecting both the stability of immobilization and subsequent release dynamics (e.g., interactions between fungal cell walls and alginate have been evidenced by spectroscopic analyses and FT-IR characterisation in encapsulated systems) (Lopes et al. 2020).

Encapsulation offers advantages such as protection of conidia against biotic and abiotic stresses and improved stability during storage and application (Ngulela et al. 2025; Pinotti et al. 2025). Among the encapsulation systems reported for microbial biocontrol agents, alginate microbeads present distinct advantages that make them particularly suitable for agricultural use. Alginate is a natural, biodegradable, non-toxic, and low-cost polymer that allows encapsulation under mild aqueous conditions, preserving microbial viability more effectively than systems based on synthetic polymers or processes requiring organic solvents or harsh conditions (Trabelsi and Mhamdi 2013; Bashan et al. 2014). In addition, alginate capsules provide a hydrated matrix that supports microbial survival and enables gradual propagule release, which can enhance persistence and efficacy in phytopathogen control.

From an industrial perspective, the high conidial viability preserved after encapsulation represents an important advantage for the large-scale production of alginate-based formulations. High survival rates during processing are directly linked to production yield and cost-effectiveness, as losses at this stage can significantly compromise the economic feasibility of biocontrol products. Nevertheless, although viability preservation suggests a favorable yield, the present study did not include quantitative assessments of production efficiency or process losses. Consequently, future scale-up studies should incorporate dedicated yield analyses and techno-economic evaluations to more accurately determine the industrial viability of this encapsulation strategy under commercial production conditions.

### Release profile of encapsulated conidia in different pH solutions

The results suggest that both pH and exposure time influence conidial release differently depending on the fungal species. In general, as capsules remained in contact with the buffer solution, conidial release tended to equilibrate across treatments, with more pronounced release observed only at higher pH values during the initial hours, a trend also reported by other authors (Adzmi 2021; Arias-Chavarría et al. 2025). This effect occurs because pH directly affects polymer degradation and hydration capacity (Vinceković et al. 2017; Wang et al. 2023).

At lower pH levels, capsules exposed to pH 5.0 and 5.5 acquired a gel-like consistency and increased in size. This behavior can be attributed to the protonation of carboxyl groups from organic acids, which enhances bonding with glucuronic acid (G-blocks) in alginate (Cruz-Barrera et al. 2024). In contrast, at higher pH values, capsules were visibly degraded within the first hours of exposure. Regarding fungal performance, conidial germination was influenced by prolonged exposure to specific pH conditions in a species-dependent manner. In particular, exposure to neutral pH appeared to impair germination in some isolates, possibly due to reduced enzymatic tolerance or structural instability of the conidial cell wall under these conditions, thereby compromising initial metabolism and germination efficiency.

Understanding the specific requirements for the use of encapsulated products is fundamental to ensuring their effectiveness in the field. Each *Trichoderma* species exhibits distinct behavior across different pH ranges, directly influencing the rate and timing of conidial release. This knowledge enables the strategic selection of the strain best adapted to the edaphoclimatic conditions of the application site. For example, *Trichoderma* sp. 1 showed maximum conidial release at pH 5.0, indicating its potential for use in acidic soils, such as those found in degraded Cerrado areas or soils with high residual acidity. In these environments, slower and more sustained release favors persistence in the rhizosphere and reduces competition with the native microbiota (Vassilev et al. 2020; Balla et al. 2022).

*T. koningiopsis and T. longibrachiatum* exhibited greater efficiency of conidial release at pH 6.5-7.0.5.0 (Fig. 4A and 4B, respectively), suggesting better adaptation to agricultural systems with limed soils, such as intensive maize and vegetable production. In these systems, faster release favors early and effective rhizosphere colonization. In addition, *Trichoderma* sp. 2 showed complete release of conidia within the first hour at pH 7.0, suggesting suitability for scenarios requiring immediate biofungicidal action, such as fields under high pathogen inoculum pressure or highly mineralized soils. *T. orientale*, in turn, displayed a stable and progressive release pattern, making it a versatile candidate across different pH ranges, provided sufficient soil exposure time is available to ensure activation.

Since maize performs well in soils with pH values between 5.5 and 7.0, with an optimum range of 6.0-6.5.0.5 (Olson and Sander 2015), it is essential that biological products used in this system are compatible with these conditions. This pH range is crucial not only for nutrient availability but also for the activity and establishment of beneficial microorganisms such as *Trichoderma*. The results of this study demonstrated that the encapsulated species remained viable and capable of releasing conidia across the pH range recommended for maize cultivation, reinforcing their agronomic applicability under diverse edaphic conditions within this production system.

### In vitro antagonistic potential against *F. verticillioides*

The maintenance of antagonistic activity after encapsulation and prolonged storage indicates that the adopted technological process preserved not only conidial viability but also the ability of the fungus to rapidly respond to the presence of the pathogen. This suggests that, once released from the alginate matrix, the encapsulated conidia were able to promptly resume essential metabolic processes, including germination, mycelial growth, and activation of antagonistic mechanisms, without significant functional delay.

The differences observed among *Trichoderma* species can be interpreted as a reflection of intrinsic physiological traits, particularly those related to colonization rate and efficiency in substrate exploitation. Isolates with higher mycelial growth capacity tend to establish spatial dominance more rapidly, thereby limiting the development of *F. verticillioides*. This behavior indicates that encapsulation did not homogenize the biological performance of the isolates but rather preserved their functional specificities.

The superior performance of *T. longibrachiatum* indicates that its antagonistic mechanisms remained operative even after the storage period, pointing to the preservation of enzymatic and metabolic activities associated with mycoparasitism and antibiosis. In this context, the alginate matrix did not act as a physiological barrier but rather as a transient microenvironment capable of protecting the conidia without compromising their capacity for metabolic activation upon exposure to the pathogen, as previously suggested by Locatelli et al. 2018 and Maruyama et al. 2020.

The pronounced performance of endophytic isolates further supports the hypothesis that ecological origin directly influences antagonistic responses after encapsulation. Isolates adapted to environments characterized by intense microbial competition tend to exhibit greater metabolic plasticity, which may explain their ability to maintain antagonistic efficacy even after the physical and chemical processes associated with formulation. This behavior is consistent with previous observations on Cerrado-derived isolates, which frequently display active secondary metabolism and rapid responses to biotic stress (Morais et al. 2022).

### In vivo growth assay

The development and sporulation of *Trichoderma* are conditioned by factors such as nutrient availability, moisture, pH, and the presence of organic compounds (Woo et al. 2014; Zehra et al. 2017). The commercial substrate, being richer in organic matter and available compounds, may have favored not only conidial germination but also mycelial growth and sporulation of the isolates, which explains the behavior observed in treatments containing commercial substrate. The observation of encapsulated conidia germinating within 24 h, followed by intense sporulation after 72 h, is consistent with studies demonstrating the rapid metabolic activation capacity of viable conidia when exposed to favorable conditions such as high moisture and organic substrates (Mukherjee et al. 2013).

The differences observed among treatments mainly reflect the interaction between the physiological characteristics of each isolate and the physicochemical conditions of the evaluated environments. The maintenance of high sporulation levels across different substrates indicates that encapsulation preserved not only viability for most isolates but also their capacity for multiplication. In this context, the attainment of propagule concentrations compatible with field application supports the potential of this technology for the development of stable biological formulations (Oancea et al. 2017; Martinez et al. 2023).

Isolates such as *Trichoderma* sp. 1 and *Trichoderma* sp. 2 may exhibit high ecological plasticity, allowing them to adapt to variations in nutrient availability and to the microbiological complexity of the environment, which explains their ability to colonize all evaluated treatments. This functional stability is particularly relevant in heterogeneous agricultural systems, where environmental fluctuations may compromise the performance of less adaptable biological control agents (Verma et al. 2007).

In contrast, the response of *T. longibrachiatum* indicates a stronger dependence on environments with more favorable biochemical conditions, including higher organic matter content, nutrient availability, and moisture. Its greater efficiency in organic-rich substrates can be attributed to a high capacity for producing hydrolytic enzymes, particularly those involved in the degradation of plant structural compounds, which enhances both growth and antagonistic activity (Sonneveld and Voogt 2009; Andrzejak and Janowska 2022; Arias et al. 2023). However, this metabolic specialization may limit its performance in nutrient-poor soils or under less conservative management systems, suggesting that its practical application may require complementary strategies, such as association with organic amendments or prior improvement of soil conditions.

The response of *T. koningiopsis* suggests a physiological limitation associated with adaptation to environments with low organic matter availability, indicating lower ecological plasticity. Its dependence on more favorable nutritional conditions for conidial germination and sporulation may hinder establishment in mineral soils, where high microbial complexity and physicochemical constraints reduce its competitiveness relative to other *Trichoderma* species. This behavior is consistent with previously reported interspecific differences in rhizosphere colonization efficiency and resource exploitation (Mukherjee et al. 2013). From an applied perspective, these results indicate that the performance of *T. koningiopsis* in encapsulated formulations may be optimized through complementary strategies, such as association with organic sources or fermented plant residues, as well as multi-strain formulations, considering its effective conidial release within the pH range of 6.5-7.0.5.0.

The behavior observed for *T. orientale* indicates that the combination of soil and commercial substrate may have created a microenvironment favorable for vegetative growth but less conducive to reproductive induction. This pattern suggests that factors related to the physicochemical composition of the medium, such as assimilable carbon availability and moisture dynamics, play a decisive role in the transition between mycelial growth and sporulation. In *Trichoderma* spp., sporulation is tightly regulated by specific environmental cues, and conditions that sustain vegetative growth do not necessarily promote reproductive differentiation (Andrzejak and Janowska 2022).

## Conclusion

Sodium alginate encapsulation effectively preserved the viability and functional stability of *Trichoderma* conidia during long-term storage at room temperature. All evaluated species maintained biologically relevant propagule levels after 15 months, indicating that the encapsulation matrix provided sufficient protection without impairing post-storage metabolic reactivation.

Conidial release from alginate capsules was modulated by pH and exposure time in a species-dependent manner, while fungal establishment and sporulation after release were further influenced by substrate physicochemical properties. Antagonistic activity against *Fusarium verticillioides* was retained after storage, although post-establishment population dynamics varied among isolates and substrates.

Overall, these results demonstrate that alginate-based encapsulation supports long-term viability, controlled conidial release, and preservation of key functional traits under controlled conditions. Future studies may evaluate capsule performance in plant-based systems inoculated with pathogens under prolonged-release conditions to directly assess conidial release dynamics and biocontrol effectiveness, thereby extending the findings of the present in vitro and in vivo substrate-based assays.

## Supplementary Information

Below is the link to the electronic supplementary material.

Supplementary Material 1 (DOCX 1.01 MB)

## Data Availability

Data will be made available on request.
