# Supplementary material for: Formulation of Trichoderma spp. encapsulated in alginate: potential for biofungicide with controlled conidial release
Source: World J Microbiol Biotechnol. 2026 May 11;42(6):277. doi: 10.1007/s11274-026-04989-9 (PMC13161278; doi:10.1007/s11274-026-04989-9)
Supplement: Supplementary file 1 — Supplementary Material 1 (DOCX 1.01 MB) [file 11274_2026_4989_MOESM1_ESM.docx]

**SUPPLEMENTARY MATERIAL**

**Formulation of *Trichoderma* spp. Encapsulated in Alginate: Potential for Biofungicide with Controlled Conidial Release**

Thalesram Izidoro Pinotti (0009-0004-5063-0606)^a*^

Vinicius de Paula Taffarel (0009-0002-8228-976X)^a^

Gabriel de Araújo Silva Cipriano (0009-0004-2637-5481)^a^

Ionnara Diogo Xavier ([0009-0005-2160-9354](https://orcid.org/0009-0005-2160-9354))^a^

Yanka Manoelly dos Santos Gaspar ([0009-0000-5855-3183](https://orcid.org/0009-0000-5855-3183))^a^

Luysa Valéria Leal Coêlho Ramos (0009-0004-8117-8771)^a^

Augusto Matias de Oliveira ([0000-0003-3556-2030](https://orcid.org/0000-0003-3556-2030))^b^

Marcos Antonio Barbosa de Lima (0000-0001-5987-224X)^c^

Galba Maria de Campos-Takaki (0000-0002-0519-0849)^d^

Tiago de Oliveira Sousa (0000-0001-7855-348X)^a^

Thiago Pajeú Nascimento (0000-0003-3480-6734)^a^

Alice Maria Gonçalves Santos (0000-0003-0672-1709)^a^

*^a^ Campus Professora Cinobelina Elvas, Federal University of Piauí, Bom Jesus, PI, 64900-000, Brazil.*

^b^ *University of Rio Verde, Rio Verde, GO, 75901-970, Brazil*

*^c^ Department of Biology, Federal Rural University of Pernambuco, Recife, PE, 52171-900, Brazil.*

*^d^ Center for Research in Environmental Sciences – Department of Chemistry, Catholic University of Pernambuco, Recife, PE, 50050-900, Brazil.*

**Corresponding author. Email address:* [thalesram@hotmail.com](mailto:thalesram@hotmail.com)

*
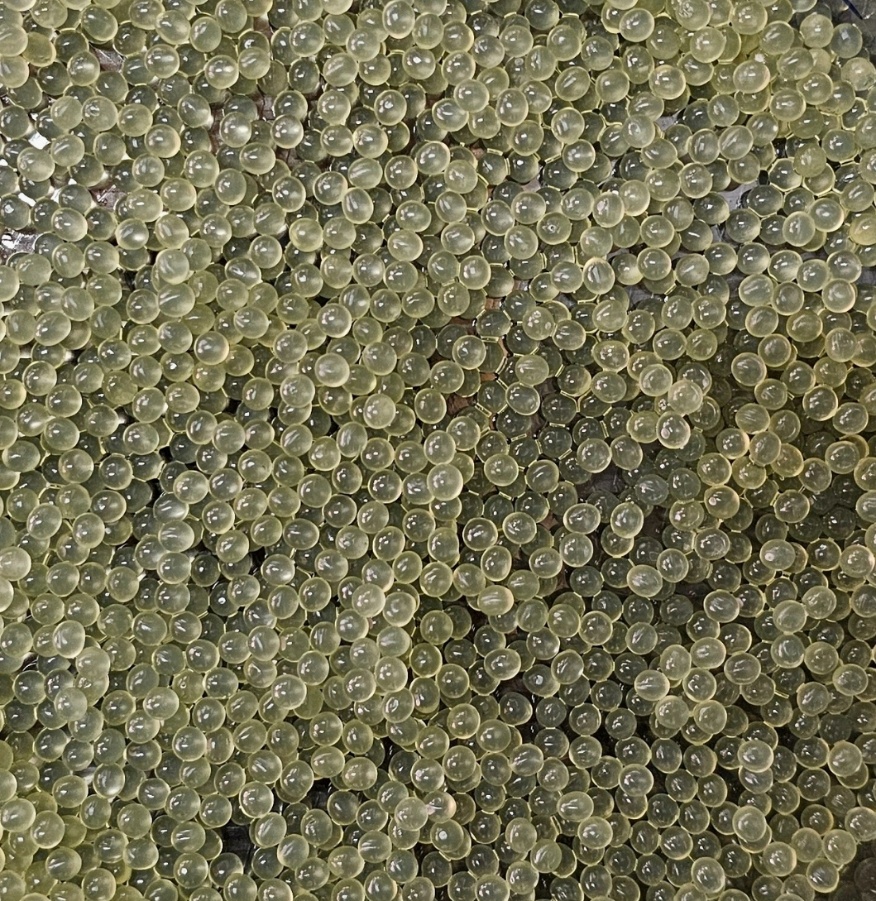
*

**Fig. S1** Representative sodium alginate capsules containing *Trichoderma* conidia after 15 months of storage

**Table S1** Analysis of variance of conidial concentration of *Trichoderma* isolates grown from alginate capsules under different storage periods

| **Species** | **Storage time (months)** | **Mean conidial concentration (CFU mL⁻¹)** | **df** | **F** | **P** | **cv (%)** |
| --- | --- | --- | --- | --- | --- | --- |
| *T* *longibrachiatum* | Before | 1.1x10^7^ | 3 | 7.7144 | 0.0003 | 24.13 |
|  | 5 | 2.8x10^7^ |  |  |  |  |
|  | 10 | 2.7x10^7^ |  |  |  |  |
|  | 15 | 2.5 x10^7^ |  |  |  |  |
| *T. koningiopsis* | Before | 1.5x10^7^ | 3 | 13.177 | 0.0004 | 24.37 |
|  | 5 | 2.6x10^7^ |  |  |  |  |
|  | 10 | 1.2x10^7^ |  |  |  |  |
|  | 15 | 1.0x10^7^ |  |  |  |  |
| *Trichoderma* sp.1 | Before | 2.4x10^7^ | 3 | 133.77 | <0.0001 | 5.47 |
|  | 5 | 3.2x10^7^ |  |  |  |  |
|  | 10 | 4.3x10^7^ |  |  |  |  |
|  | 15 | 5.1x10^7^ |  |  |  |  |
| *Trichoderma* sp.2 | Before | 2.0x10^7^ | 3 | 91.36 | <0.0001 | 15.17 |
|  | 5 | 3.9x10^7^ |  |  |  |  |
|  | 10 | 8.0x10^7^ |  |  |  |  |
|  | 15 | 1.3x10^8^ |  |  |  |  |
| *T. orientale* | Before | 1.4x10^7^ | 3 | 28.013 | <0.0001 | 6.22 |
|  | 5 | 1.0x10^7^ |  |  |  |  |
|  | 10 | 1.0x10^7^ |  |  |  |  |
|  | 15 | 1.0x10^7^ |  |  |  |  |

df: degrees of freedom; cv: coefficient of variation.

**Table S2** Analysis of variance of the antagonistic effect of microencapsulated *Trichoderma longibrachiatum* under different pH solutions and agitation times

| **SV** | **df** | **SS** | **MS** | **Fc** | **Pr>Fc** |
| --- | --- | --- | --- | --- | --- |
| pH | 4 | 2005350000 | 501337500 | 215.63 | 0.0000 |
| Hours (h) | 2 | 5342925000 | 2671462500 | 1149.02 | 0.0000 |
| pH * h | 8 | 719950000 | 89993750 | 38.71 | <0.0001 |
| Residual | 45 | 104625000 | 2325000 |  |  |
| Total | 59 | 8172850000 | 138522881 |  |  |
| cv (%) = | 5.76 | | | | |
| **UNFOLDING OF THE INTERACTION EFFECT** | | | | | |
| SV | df | SS | MS | Fc | Pr>Fc |
| pH/1 h | 4 | 1926125000 | 481531250 | 207.1102 | 0.0000 |
| pH/5 h | 4 | 102925000 | 25731250 | 11.0672 | 0.0000 |
| pH/24 h | 4 | 696250000 | 174062500 | 11.0672 | 0.0000 |
| Residual | 45 | 104625000 | 2325000 |  |  |
| SV | df | SS | MS | Fc | Pr>Fc |
| h/pH 5.0 | 2 | 1658375000 | 829187500 | 356.6398 | 0.0000 |
| h/pH 5.5 | 2 | 1623166667 | 811583333 | 349.0681 | 0.0000 |
| h/pH 6.0 | 2 | 1776166667 | 888083333 | 381.9713 | 0.0000 |
| h/pH 6.5 | 2 | 241291667 | 120645833 | 51.8907 | 0.0000 |
| h/pH 7.0 | 2 | 763875000 | 381937500 | 164.2742 | 0.0000 |
| Residual | 45 | 104625000 | 2325000 |  |  |

SV: source of variation; df: degrees of freedom; SS: sum of squares; MS: mean square; cv: coefficient of variation.

**Table S3** Analysis of variance of the antagonistic effect of microencapsulated *Trichoderma koningiopsis* under different pH solutions and agitation times

| **SV** | **df** | **SS** | **MS** | **Fc** | **Pr>Fc** |
| --- | --- | --- | --- | --- | --- |
| pH | 4 | 2067941667 | 516985416.8 | 212.58 | 0.0000 |
| Hours (h) | 2 | 5515108333 | 2757554167 | 1133.89 | 0.0000 |
| pH * h | 8 | 712558333 | 89069791.63 | 36.62 | <0.0001 |
| Residual | 45 | 109437500 | 2431944 |  |  |
| Total | 59 | 8405045833 | 142458403.9 |  |  |
| cv (%) = | 5.9 | | | | |
| **UNFOLDING OF THE INTERACTION EFFECT** | | | | | |
| SV | df | SS | MS | Fc | Pr>Fc |
| pH/1 h | 4 | 1962300000 | 490575000 | 201.7213 | 0.0000 |
| pH/5 h | 4 | 131125000 | 32781250 | 13.4794 | 0.0000 |
| pH/24 h | 4 | 687075000 | 17176875 | 70.6302 | 0.0000 |
| Residual | 45 | 109437500 | 2431944 |  |  |
| SV | df | SS | MS | Fc | Pr>Fc |
| h/pH 5.0 | 2 | 1769291667 | 884645833 | 363.7607 | 0.0000 |
| h/pH 5.5 | 2 | 1621541667 | 810770833 | 333.3838 | 0.0000 |
| h/pH 6.0 | 2 | 1812791667 | 906395833 | 372.7042 | 0.0000 |
| h/pH 6.5 | 2 | 257541667 | 128770833 | 52.9497 | 0.0000 |
| h/pH 7.0 | 2 | 766500000 | 383250000 | 157.5899 | 0.0000 |
| Residual | 45 | 109437500 | 2431944 |  |  |

SV: source of variation; df: degrees of freedom; SS: sum of squares; MS: mean square; cv: coefficient of variation.

**Table S4** Analysis of variance of the antagonistic effect of microencapsulated *Trichoderma* sp.1 under different pH solutions and agitation times

| **SV** | **df** | **SS** | **MS** | **Fc** | **Pr>Fc** |
| --- | --- | --- | --- | --- | --- |
| pH | 4 | 1.8177e+08 | 45442500 | 11.13 | <0.0001 |
| Hours (h) | 2 | 1.0240e+10 | 5120000000 | 1253.48 | 0.0000 |
| pH * h | 8 | 1.9472e+09 | 243400000 | 59.59 | 0.0000 |
| Residual | 45 | 1.8381e+08 | 4084666.667 |  |  |
| Total | 59 | 1.2553e+10 | 212762711.9 |  |  |
| cv (%) = | 7.08 | | | | |
| **UNFOLDING OF THE INTERACTION EFFECT** | | | | | |
| SV | df | SS | MS | Fc | Pr>Fc |
| pH/1 h | 4 | 436175000 | 109043750 | 26.6955 | 0.0000 |
| pH/5 h | 4 | 69175000 | 17293750 | 4.2338 | 0.0054 |
| pH/24 h | 4 | 1623675000 | 405918750 | 99.3749 | 0.0000 |
| Residual | 45 | 183812500 | 4084722 |  |  |
| SV | df | SS | MS | Fc | Pr>Fc |
| h/pH 5.0 | 2 | 5028041667 | 2514020833 | 615.4692 | 0.0000 |
| h/pH 5.5 | 2 | 2988541667 | 1494270833 | 365.8194 | 0.0000 |
| h/pH 6.0 | 2 | 2298041667 | 1149020833 | 281.2972 | 0.0000 |
| h/pH 6.5 | 2 | 863291667 | 431645833 | 105.6732 | 0.0000 |
| h/pH 7.0 | 2 | 1009541667 | 504770833 | 123.5753 | 0.0000 |
| Residual | 45 | 183812500 | 4084722 |  |  |

SV: source of variation; df: degrees of freedom; SS: sum of squares; MS: mean square; cv: coefficient of variation.

**Table S5** Analysis of variance of the antagonistic effect of microencapsulated *Trichoderma* sp.2 under different pH solutions and agitation times

| **SV** | **df** | **SS** | **MS** | **Fc** | **Pr>Fc** |
| --- | --- | --- | --- | --- | --- |
| pH | 4 | 676191667 | 169047916.8 | 21.558 | <0.0001 |
| Hours (h) | 2 | 3080633333 | 1540316667 | 196.427 | 0.0000 |
| pH * h | 8 | 1090033333 | 136254166.6 | 17.376 | <0.0001 |
| Residual | 45 | 352875000 | 7841666.667 |  |  |
| Total | 59 | 5199733333 | 88131073.44 |  |  |
| cv (%) = | 4.12 | | | | |
| **UNFOLDING OF THE INTERACTION EFFECT** | | | | | |
| SV | df | SS | MS | Fc | Pr>Fc |
| pH/1 h | 4 | 1727825000 | 431956250 | 55.0848 | 0.0000 |
| pH/5 h | 4 | 36075000 | 9018750 | 1.1501 | 0.3454 |
| pH/24 h | 4 | 2325000 | 581250 | 0.0741 | 0.9897 |
| Residual | 45 | 352875000 | 7841667 |  |  |
| SV | df | SS | MS | Fc | Pr>Fc |
| h/pH 5.0 | 2 | 1920666667 | 960333333 | 122.4655 | 0.0000 |
| h/pH 5.5 | 2 | 1193166667 | 596583333 | 76.0786 | 0.0000 |
| h/pH 6.0 | 2 | 926166667 | 463083333 | 59.0542 | 0.0000 |
| h/pH 6.5 | 2 | 92666667 | 46333333 | 5.9086 | 0.0053 |
| h/pH 7.0 | 2 | 38000000 | 19000000 | 2.423 | 0.1001 |
| Residual | 45 | 352875000 | 7841667 |  |  |

SV: source of variation; df: degrees of freedom; SS: sum of squares; MS: mean square; cv: coefficient of variation.

**Table S6** Analysis of variance of the antagonistic effect of microencapsulated *Trichoderma* *orientale* under different pH solutions and agitation times

| **SV** | **df** | **SS** | **MS** | **Fc** | **Pr>Fc** |
| --- | --- | --- | --- | --- | --- |
| pH | 4 | 3.7065e+09 | 926625000 | 143.69 | <0.0001 |
| Hours (h) | 2 | 1.4728e+10 | 7364000000 | 1141.96 | 0.0000 |
| pH * h | 8 | 4.5155e+09 | 564437500 | 87.53 | <0.0001 |
| Residual | 45 | 2.9019e+08 | 6448666.667 |  |  |
| Total | 59 | 2.3240e+10 | 393898305.1 |  |  |
| cv (%) = | 5.67 | | | | |
| **UNFOLDING OF THE INTERACTION EFFECT** | | | | | |
| SV | df | SS | MS | Fc | Pr>Fc |
| pH/1 h | 4 | 7716300000 | 1929075000 | 299.1458 | 0.0000 |
| pH/5 h | 4 | 502875000 | 125718750 | 19.4955 | 0.0000 |
| pH/24 h | 4 | 2825000 | 706250 | 0.1095 | 0.9786 |
| Residual | 45 | 290187500 | 6448611 |  |  |
| SV | df | SS | MS | Fc | Pr>Fc |
| h/pH 5.0 | 2 | 6987541667 | 3493770833 | 541.7866 | 0.0000 |
| h/pH 5.5 | 2 | 5741166667 | 2870583333 | 445.1475 | 0.0000 |
| h/pH 6.0 | 2 | 5064291667 | 2532145833 | 392.6653 | 0.0000 |
| h/pH 6.5 | 2 | 1430541667 | 715270833 | 110.9186 | 0.0000 |
| h/pH 7.0 | 2 | 20041667 | 10020833 | 1.554 | 0.2225 |
| Residual | 45 | 290187500 | 6448611 |  |  |

SV: source of variation; df: degrees of freedom; SS: sum of squares; MS: mean square; cv: coefficient of variation.

**Table S7** Analysis of variance of the antagonistic effect of microencapsulated *T. longibrachiatum* (T1), *T.* *koningiopsis* (T2), *Trichoderma* sp.1 (T3), *Trichoderma* sp.2 (T4), and *T. orientale* (T5) on the final colony diameter (FCD) and percentage of growth inhibition (PGI) of *Fusarium verticillioides* (Control)

| **Variable** | **Treatments** | **Mean** | **df** | **F** | **P** | **cv (%)** |
| --- | --- | --- | --- | --- | --- | --- |
| FDC | T1 | 33.26 | 5 | 65.974 | <0.0001 | 5.96 |
|  | T2 | 45.66 |  |  |  |  |
|  | T3 | 39.30 |  |  |  |  |
|  | T4 | 37.85 |  |  |  |  |
|  | T5 | 36.57 |  |  |  |  |
|  | Control | 61.56 |  |  |  |  |
| MGI | T1 | 45.97 | 5 | 94.049 | <0.0001 | 11.02 |
|  | T2 | 25.82 |  |  |  |  |
|  | T3 | 36.15 |  |  |  |  |
|  | T4 | 38.51 |  |  |  |  |
|  | T5 | 40.58 |  |  |  |  |
|  | Control | 0.0 |  |  |  |  |

df: degrees of freedom; cv: coefficient of variation.


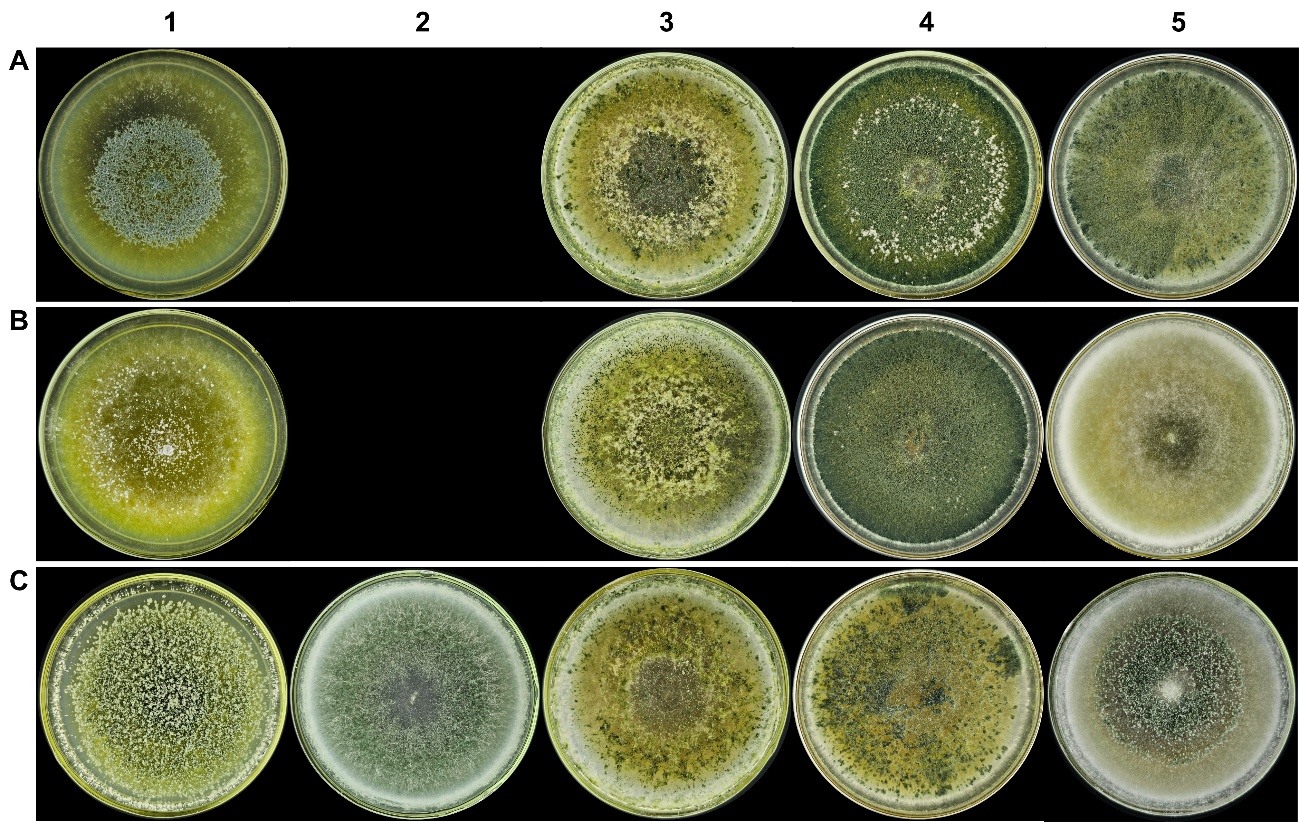


**Fig. S2** Growth of *T. longibrachiatum* (1), *T. koningiopsis* (2), *Trichoderma* sp.1 (3), *Trichoderma* sp.2 (4), and *T. orientale* (5) reisolated from growth media: soil (A), soil + commercial substrate (B), and commercial substrate (C). For *T. koningiopsis* (2), images from treatments A and B are not shown because the fungus did not grow under these conditions

**Table S8** Analysis of variance of conidial concentration of *T. longibrachiatum* (A), *T. koningiopsis* (B), *Trichoderma* sp.1 (C), *Trichoderma* sp.2 (D), and *T. orientale* (E) reisolated from different treatments

| **Species** | **Treatments** | **Mean** | **df** | **F** | **P** | **cv (%)** |
| --- | --- | --- | --- | --- | --- | --- |
| *T. longibrachiatum* | Soil | 1x10^7^ | 3 | 143.49 | <0.0001 | 18.56 |
|  | Soil + Substrate | 1x10^6^ |  |  |  |  |
|  | Substrate | 6x10^7^ |  |  |  |  |
|  | Reference value | 2x10^7^ |  |  |  |  |
| *T. koningiopsis* | Substrate | 1x10^7^ | 3 | 28.484 | <0.0001 | 43.27 |
|  | Reference value | 1x10^7^ |  |  |  |  |
| *Trichoderma* sp.1 | Soil | 5x10^7^ | 3 | 2.2521 | <0.0001 | 3.55 |
|  | Soil + Substrate | 5x10^7^ |  |  |  |  |
|  | Substrate | 5x10^7^ |  |  |  |  |
|  | Reference value | 5x10^7^ |  |  |  |  |
| *Trichoderma* sp.2 | Soil | 1x10^8^ | 3 | 50.827 | <0.0001 | 8.99 |
|  | Soil + Substrate | 1x10^8^ |  |  |  |  |
|  | Substrate | 5x10^7^ |  |  |  |  |
|  | Reference value | 1x10^8^ |  |  |  |  |
| *T. orientale* | Soil | 6x10^7^ | 3 | 488.94 | <0.0001 | 8.91 |
|  | Soil + Substrate | 0 |  |  |  |  |
|  | Substrate | 5x10^7^ |  |  |  |  |
|  | Reference value | 1x10^7^ |  |  |  |  |

df: degrees of freedom; cv: coefficient of variation.
